# Invisible Gorillas in the Mind: Internal Inattentional Blindness and the Prospect of Introspection Training

**DOI:** 10.1162/opmi_a_00204

**Published:** 2025-04-22

**Authors:** Adam Morris

**Affiliations:** Psychology Department, Princeton University, Princeton, NJ, USA

**Keywords:** consciousness, internal attention, inattentional blindness, introspection, mindfulness, preconscious, self-awareness, mental training

## Abstract

Much of high-level cognition appears inaccessible to consciousness. Countless studies have revealed mental processes—like those underlying our choices, beliefs, judgments, intuitions, etc.—which people do not notice or report, and these findings have had a widespread influence on the theory and application of psychological science. However, the interpretation of these findings is uncertain. Making an analogy to perceptual consciousness research, I argue that much of the unconsciousness of high-level cognition is plausibly due to *internal inattentional blindness*: missing an otherwise consciously-accessible internal event because your attention was elsewhere. In other words, rather than being structurally unconscious, many higher mental processes might instead be “preconscious”, and would become conscious if a person attended to them. I synthesize existing indirect evidence for this claim, argue that it is a foundational and largely untested assumption in many applied interventions (such as therapy and mindfulness practices), and suggest that, with careful experimentation, it could form the basis for a long-sought-after science of introspection training.

## INTRODUCTION

Humans have been trying to introspect on their mental processes for a long time. Eastern contemplatives strived for millennia to become aware of what was happening inside themselves through meditative training (Shear & Jevning, 1999); in Western psychology, introspectionists like Wundt spent thousands of hours training participants to observe the workings of their own mind (Cowan & Rachev, 2018; Schwitzgebel, 2004), and early psychoanalysts like Anna Freud claimed that a central goal of therapy was to “bring into consciousness that which is unconscious” (quoted in Wilson, 2004, p. 15). In contemporary society, widespread methods for mental improvement—such as cognitive therapies, mindfulness practices, and many popular personal/professional development techniques—purport to train awareness of the processes underlying one’s judgments, choices, and feelings (Dahl et al., 2015, 2020).

At the same time, modern experimental psychology has uncovered an enormous amount of high-level processes to which people do not seem to have introspective access. Starting with Nisbett and Wilson (1977), experiments have revealed fundamental unconscious processes underlying choice, judgment, attitudes, beliefs, and just about every other part of human experience (Bargh & Morsella, 2008; Evans, 2008; Gigerenzer, 2008; Haidt, 2001; Hassin et al., 2006; Kahneman, 2011). These data are often interpreted as showing that people cannot reliably introspect on the workings of their minds. For instance, in Wilson’s (2004) influential book *Strangers to Ourselves*, he writes (p. 16):

It is difficult to know ourselves because there is no direct access to the adaptive unconscious, no matter how hard we try. Because our minds have evolved to operate largely outside of consciousness, and nonconscious processing is part of the architecture of the brain, it may not be possible to gain direct access to nonconscious processes.

On this perspective, people can, at best, describe the current contents of their subjective experience or working memory (Dehaene, 2014; Ericsson & Simon, 1980), the outputs of their mental processes (Nisbett & Wilson, 1977), or some abstracted features of those processes (like their overall accuracy; Fleming et al., 2012)—but, since so much of cognition happens unconsciously, most detailed reports of the processes underneath these surface-level experiences are untrustworthy (Wilson, 2004).^1^ There is a tension between this view—that an enormous percentage of high-level cognition occurs unconsciously, and cannot be accessed introspectively—and the persistent societal belief that people can acquire substantial, process-level self-awareness through training.

I argue that this tension results from a hypothesis which is confidently assumed by practitioners and rarely explored by experimental scientists: that some unconscious processes are unconscious only because of inattention. This hypothesis, which I call *internal inattentional blindness*, states that these unconscious processes are not *inherently* unconscious; their unconsciousness is not a structural or static feature of the mind. Rather, they are unconscious for the same reason that the gorilla in Simons and Chabris’s (1999) classic study was unconscious: People fail to pay attention to them. If people did pay attention to the processes, those processes would become conscious—and hence people can learn, through internal attentional training, to become aware of many of the mental processes underlying their experience and behavior.

In this paper, I lay out the internal inattentional blindness (IIB) hypothesis in detail and review existing evidence for it, of which there is much indirect and very little direct. I then sketch a roadmap for testing the hypothesis directly, and discuss its ramifications for both applied and basic psychology. The ramifications for applied psychology are clear: Since (as argued below) the hypothesis is widely assumed in popular applied frameworks, testing it directly would either put these frameworks on more solid scientific ground, or suggest that they need major revision.

The possibility of bringing unconscious processes into awareness also has significant implications for basic, experimental psychology. The scope of conscious access to one’s own mental processes is a fundamental question about the mind. Though there has been much debate about which processes are *inherently* conscious or unconscious (Bargh & Morsella, 2008; Chater, 2018; Ericsson & Simon, 1984; Newell & Shanks, 2014; Smith & Miller, 1978), the idea that unconscious processes could be raised into awareness has not been systematically explored (Locke, 2009; Schwitzgebel, 2004), in part because there has not been a cognitively-grounded theory of how such a raising could occur. IIB offers such a theory. If it is right, the IIB hypothesis would challenge the long-standing conclusion that “there is no direct access to the adaptive unconscious, no matter how hard we try” (Wilson, 2004), and suggest that the observed ubiquity of unconscious processes in high-level cognition may not be a structural constraint of the mind, but rather an incidental reflection of internal inattention.

Finally, if the IIB hypothesis is right, it could offer a means of accelerating the pace of psychological research. Psychological science is, fundamentally, reverse engineering: Examining the mind from the outside, as one would an unfamiliar object (Pinker, 1997). While introspection should of course not replace objective data as the measuring stick of psychological truth, a training that verifiably improves people’s ability to access their own mental processes could dramatically speed up this reverse engineering process—by guiding hypothesis generation, theory building, and experimental design down more profitable paths (Jack & Roepstorff, 2003). Dehaene (2014) writes: “Obviously we cannot count on naive human subjects to tell us how their mind works; otherwise our science would be too easy”. This may be true. But if trained subjects can actually, to some extent, tell us how their mind works, our science would plausibly get easier.

## CONCEPTUALIZING INTERNAL INATTENTIONAL BLINDNESS

### Inattentional Blindness Towards External Perceptual Stimuli

Theories of inattentional blindness are built on two fundamental cognitive constructs: attention and conscious awareness. Though the two constructs are notoriously difficult to distinguish and define, attention has to do with the selection or prioritization of information for processing; when there are multiple things (e.g., visual stimuli) competing for limited-capacity processing, the mind prioritizes some at the expense of others, and the mechanisms that support this capacity are what constitute attention (Chun et al., 2011; Cohen et al., 2012). Attention is often characterized as a gatekeeper (Awh et al., 2006), letting some information in for processing and excluding the rest.

In contrast, conscious awareness (in the sense I will focus on here) has to do with information becoming widely available across cognition to the mind’s “consumer systems” like verbal report, reasoning, rational control of action, and so on (Baars, 2002; Block, 1995, 2005; Dehaene & Naccache, 2001). Information that fails to enter consciousness (e.g., a subliminally-flashed word) can narrowly impact future cognition, through domain-specific peripheral processing or through effects like priming; but information that enters consciousness (e.g., the words you’re reading right now) becomes globally available for report, inference, decision-making, recollection, and other high-level mental functions (Dehaene, 2014). This sense of conscious awareness has been called “access consciousness” (Baars, 2002; Block, 2005; Dehaene & Naccache, 2001), and has been associated with information being maintained in a long-range, brain-wide recurrent neural network (referred to as a “global neuronal workspace”; Dehaene et al., 2011). One simplified way to put it is that consciousness is a cognitive workspace which attention lets information into; consciousness is the destination, attention its gatekeeper. (Of course, there are other notions and theories of consciousness (Block, 2007; Brown et al., 2019; Lamme, 2006; Lau & Rosenthal, 2011; Tononi et al., 2016). I adopt access consciousness and global workspace theory here because they provide a natural framing for the IIB hypothesis, but IIB is also naturally compatible with other accounts; see Conceptual Clarifications for the IIB Hypothesis section for discussion.)

The precise relation between attention and conscious awareness has been the subject of heated debate, with open questions about whether attention is actually necessary or sufficient for consciousness (Cohen et al., 2012; De Brigard & Prinz, 2010; Dehaene et al., 2006; Koch & Tsuchiya, 2007; Lamme, 2003; van Boxtel et al., 2010). But there is clear evidence for one strand of this relationship: Inattention can cause otherwise-reportable perceptual stimuli to fail to enter awareness.

This fact is vividly demonstrated by “inattentional blindness” paradigms, in which people engaged in an attentionally-demanding task fail to notice salient unexpected stimuli (Mack & Rock, 1998). Famously, people fail to notice a gorilla walking unexpectedly through a visual scene if their attention is preoccupied (Drew et al., 2013; Neisser & Becklen, 1975; Simons & Chabris, 1999). People also fail to notice a distinctive shape appearing unexpectedly at fixation during a perceptual detection task (Mack & Rock, 1998; Most et al., 2005); a tone being played in their ear while they’re under visual load (Macdonald & Lavie, 2011); or a clown unicycling across their path while they’re talking on a cell phone (Hyman et al., 2010). Of course, when people are paying attention, they easily notice all these stimuli. This phenomenon of inattentional blindness has been replicated and extended many times (Bredemeier & Simons, 2012; Memmert, 2006; Richards et al., 2010; Seegmiller et al., 2011; Simons & Jensen, 2009), and has been called “one of psychology’s biggest exports” (Ward & Scholl, 2015).

These experiments—along with many others, such as work on the attentional blink (Dux & Marois, 2009; Raymond et al., 1992; Shapiro et al., 1997), hemispatial neglect/visual extinction (Duncan et al., 1997; Posner et al., 1984; Smania et al., 1998; Vuilleumier & Rafal, 2000), and change blindness (Rensink, 2002; Simons & Rensink, 2005)—suggest that there are multiple reasons a stimulus can be unconscious (Dehaene, 2014; Dehaene et al., 2006). Some stimuli are unconscious because of their intrinsic, structural features—e.g., a masked stimulus flashed for ten milliseconds might be too weak to fully reach awareness. But other stimuli fail to reach awareness because of incidental features of the observer at the time of perception—namely, the observer was not attending to them. The former stimuli are in some sense permanently unconscious, but the latter could be brought into awareness with attentional control.

**Figure 1.**
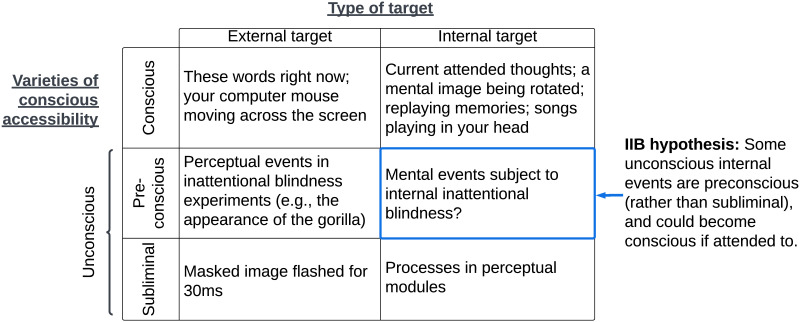
Varieties of conscious accessibility for external and internal targets. The tripartite distinction between conscious, preconscious, and subliminal was proposed by Dehaene et al. (2006), and some form of this taxonomy is now widely adopted in research on access consciousness (Dehaene, 2014). Though this distinction (and the concept of preconsciousness) has been applied almost exclusively to external, perceptual targets, it may apply to internal targets as well. The internal inattentional blindness (IIB) hypothesis is that some unconscious mental events (e.g., the activation and transformation of key representations during an unconscious decision process) are actually preconscious due to inattention, and could become conscious if attended to.

This observation is far from new, and features prominently in contemporary global workspace theories of consciousness (Figure 1). In addition to distinguishing between conscious and subliminal (i.e., too weak to ever enter consciousness) stimuli, Dehaene et al. (2006) articulate an intermediate category: stimuli which were strong enough to enter consciousness, but did not because they were not attended to. Dehaene et al. (2006) refer to these stimuli as “preconscious” (Figure 1). The notion of preconsciousness plays a fundamental role in contemporary understandings of perceptual consciousness (Dehaene, 2014; Schooler, 2002b).

An important caveat here is that it is unclear whether preconscious stimuli (like the invisible gorilla) are genuinely unconscious before being attended to—or whether instead people see them but don’t encode them in memory (“inattentional amnesia”; Wolfe, 1999) or fail to perceptually or cognitively elaborate on them (“inattentional agnosia”; agnosia”; Simons, 2000). These possibilities can be difficult to disentangle (Ward & Scholl, 2015) debate is ongoing (Nartker et al., 2025).^2^ As argued below, the thrust of this paper does not hinge on which of these interpretations is correct. I will argue that, in whatever way inattention hinders or degrades awareness of external, perceptual targets, it might similarly degrade awareness of internal, mental targets. For simplicity, I will continue to refer to this phenomenon as inattentional blindness; but the reader should keep in mind that the unattended-to targets might not be invisible, just fuzzy or forgotten.

### Inattentional Blindness Towards Internal Events

Inattentional blindness (and the existence of preconscious representations) has been studied primarily in the external domain: people failing to become aware of perceptual stimuli because their attention is elsewhere. It has not, however, been systematically applied to the domain of *internal* attention.

As opposed to external attention, which operates over perceptual representations, internal attention is the selection or prioritization for processing of internal, cognitive representations: memories, task sets, goals, judgments, beliefs, decision options, plans, mental images, and so on (Amir & Bernstein, 2022; Chun et al., 2011; De Brigard, 2012; Dixon et al., 2014; Lückmann et al., 2014). The boundaries of what exactly to characterize as internal attention are unclear, but plausible examples include selecting among competing task sets or goals (Chun et al., 2011; Gehring et al., 2003); scrutinizing features of a mental image held in working memory (Fan & Turk-Browne, 2013; Griffin & Nobre, 2003; Souza & Oberauer, 2016); retrieving an item from long-term memory (Chun & Johnson, 2011; De Brigard, 2012; Logan et al., 2021); and, more speculatively, focusing on ongoing thoughts and cognitive operations (Fortney, 2020). Internal attention is similar to the executive controller posited in working memory models, although the precise relationship between these constructs is debated (Amir & Bernstein, 2022; Amir et al., 2021; Awh et al., 2006; Chun, 2011; Kiyonaga & Egner, 2013; Lewis-Peacock et al., 2012; Myers et al., 2017). And though internal attention is dissociable from external attention, the two exhibit many similarities: they can overlap neurally (Kiyonaga & Egner, 2013), mutually interfere with each other (Kiyonaga & Egner, 2013), control which features of a representation get committed to memory (Fan & Turk-Browne, 2013), and be either intentionally controlled or “captured” by bottom-up salience (van Ede et al., 2020).

It is plausible, then, that just as inattentional blindness happens externally, it happens internally too; just as a person can fail to observe an unattended gorilla, she can also fail to observe internal events (like those preceding a judgment or decision) because she was not attending to them. In other words, internal events could be preconscious: currently unconscious, but poised to enter awareness if attended to (Figure 1).^3^

What constitutes “internal events”, and how do they relate to mental processes? I use the term internal (or mental) event to describe the events which compose symbolic mental processes: the creation or activation of representations, and the operations which transform them into other representations or behavioral outputs.^4^ For instance, consider the availability heuristic (Tversky & Kahneman, 1973). When a person uses the availability heuristic to judge the relative frequency of, e.g., homicides, they try to call to mind examples of homicide; they represent the number of examples they called to mind, or the ease with which they could generate examples (Schwarz et al., 1991; Schwarz & Vaughn, 2002); and then they integrate that information with other background knowledge to produce a frequency estimate. These mental events combine to form the process which we label the availability heuristic.

For any mental process such as this, a person could, in principle, be conscious or not of each of its component events. In practice, of course, people are often surprisingly unable to report the mental processes underlying their judgments, decisions, beliefs, attitudes, and so on (Hassin et al., 2006; Nisbett & Wilson, 1977), leading many to conclude that we are “strangers to ourselves” (Wilson, 2004). The central claim of this paper is that some of those processes, or some component events of those processes, are plausibly like invisible gorillas—not structurally unconscious, just unattended to.

This claim merits two clarifications. First: Just as inattentional “blindness” might actually be amnesia or agnosia, the same is true for *internal* inattentional blindness. Rather than unattended mental processes being entirely unconscious, it is possible that those processes had instead just been dimly conscious but forgotten, or insufficiently elaborated on. The thrust of this paper does not hinge on these distinctions. The essence of the IIB hypothesis is that whatever prevents unattended perceptual events from reaching full awareness—whether that’s blindness, amnesia, or insufficient elaboration—an analogous mechanism might also explain people’s unawareness of many of their internal mental processes (Hassin et al., 2006; Nisbett & Wilson, 1977; Pohl, 2016).

Second: The claim is not that internal attention can illuminate *all* elements of a mental process, at all levels of resolution. Mental processes are hierarchical, with high-level operations implemented in lower level computations (Badre, 2008; Botvinick, 2008; Lashley, 1951; Marr, 2010). For instance, the high-level operations composing the availability heuristic, like “call to mind examples of homicide”, are implemented in lower-level algorithms, e.g., for memory retrieval. The mental events that could most plausibly be brought into awareness are ones higher up in the hierarchy—events at the level of “call to mind homicide examples” or “represent the ease of generating examples”, not the lower-level algorithms carrying out those operations. (Although, if the IIB hypothesis is correct, then for any mental process it would be an empirical question how much of the hierarchy people can consciously traverse; see Conceptual Clarifications for the IIB Hypothesis section for discussion). I will use the language “awareness of a mental process” to mean awareness of the process’s key component events, even if those events are represented at a relatively high level in the abstraction hierarchy. Even if people can only become aware of high-level components of their (previously-unconscious) mental processes, this finding would still challenge many contemporary pictures of the mind (Bargh & Morsella, 2008; Haidt, 2001; Hassin et al., 2006; Kahneman, 2011; Wilson, 2004), and would have substantial ramifications for the theory and practice of psychology.^5^

## CIRCUMSTANTIAL EVIDENCE FOR THE IIB HYPOTHESIS

There is much circumstantial evidence suggesting that some unconscious mental processes can be brought into awareness via internal attention. This evidence is not intended as a strong scientific case for the existence of IIB; in How to Test the IIB Hypothesis section, I will lay out what rigorous experimental tests of IIB might look like, and propose a roadmap for investigating it directly. Rather, this circumstantial evidence motivates IIB as a hypothesis to pursue in the first place.

### Self-Reports From Applied Psychological Practices

The largest source of circumstantial evidence comes from applied psychological practices like therapy and mindfulness/meditation. In many of these practices, IIB is a foundational assumption, often so assumed that it is not explicitly named.

For instance, consider cognitive-behavioral therapy (CBT). CBT is a family of interventions considered the gold standard for evidence-based therapy (Hayes & Hofmann, 2017), and one of its focal points is helping people identify and reshape maladaptive thought patterns or schemas (Beck et al., 1979; Hofmann et al., 2012). A core assumption in CBT is that automatic thoughts (like the activation of negative schemas) play an important role in the mental processes underlying emotional reactions, choices, judgments, etc; that these thoughts are normally unconscious; and that they can be brought into consciousness by attending to them, through conversation with a therapist and through practices like thought journaling or “attentional control training” (Beck, 1991; Beck & Haigh, 2014; DeRubeis et al., 2010; Segal et al., 2018). Beck et al. (1979), in a foundational text on CBT, wrote:

Patients experienced specific types of thoughts of which they were only dimly aware and that they did not report. … Unless they were directed to focus their attention on these thoughts, they were not likely to be very aware of them. Although these thoughts seemed to be on the periphery of the patients’ stream of consciousness, they appeared to play an important role in the psychic life of these patients. … It seemed to me that I had tapped another level of consciousness in the recognition of automatic thoughts, perhaps analogous to the phenomenon described by Freud as “preconscious” …

Similar observations can be found in mindfulness-based cognitive therapy (Segal et al., 2018), dialectical behavioral therapy (Linehan, 1993), and many other modern therapies (Castonguay & Hill, 2006; Frank & Frank, 1993; Grant et al., 2002; Grosse Holtforth et al., 2007; Stein & Grant, 2014; Timulak & McElvaney, 2013). Pull out a therapy book, and it will likely be replete with examples of coming to notice important internal events that were going unnoticed before. The idea is so commonplace that it is rarely even presented as an important claim. Of course, not all approaches to therapy involve this kind of self-awareness (Wampold et al., 2007), and therapies differ enormously in what they say should be done with the thoughts once they are noticed; but for many modern therapies, overcoming IIB is supposed to be a critical component.

IIB is also a foundational assumption in many mindfulness and meditation practices. For instance, popular meditation teacher Joseph Goldstein writes (Goldstein, 2017, p. 18):

What happens as the mind becomes silent and we become more finely aware, is that many of the things which were below our normal threshold of awareness, much of what is called subconscious material, become illuminated by mindfulness.

Similarly, Kabat-Zinn (1994), who popularized the term “mindfulness”, argued that inattention leads to “a lack of awareness and understanding of our own mind and how it influences our perceptions and our actions”, and that mindfulness practice “literally allows us to see more clearly” into our minds (pp. 8–17).

Scientific treatments of mindfulness make similar claims. In their seminal paper, Brown and Ryan (2003) characterize mindfulness as “‘the clear and single-minded awareness of what actually happens to us and in us.’ … Rather than generating mental accounts about the self, mindfulness ‘offer[s] a bare display of what is taking place’” (Shear & Jevning, 1999; Thera, 1972). In another influential paper, Bishop et al. (2004) argue that a core component of mindfulness is “self-regulation of attention so that it is maintained on immediate experience, thereby allowing for increased recognition of mental events in the present moment.” These sentiments are echoed in other theoretical accounts of mindfulness (Dahl et al., 2015; Hadash & Bernstein, 2019; Lutz et al., 2015; Vago & David, 2012). Moreover, self-report measures of mindfulness typically include an assessment of how well someone can “observe, notice, or attend to a variety of stimuli, including internal phenomena, such as bodily sensations, cognitions, and emotions” (Baer et al., 2004, 2008; Young, 2016), and the practices often involve focusing attention on internal events for long periods of time (Goldstein, 2017; Young, 2016).

This belief in IIB also extends outside formal therapy or mindfulness practice. It appears routinely, for instance, in coaching and professional development frameworks, like Google’s popular “Search Inside Yourself” program (whose curriculum includes self-awareness training meant to “enhance your perception of your own emotions” and help you “accurately assess your thoughts”; Caporale-Berkowitz et al., 2021; Search-Inside-Yourself, 2020), or the influential “immunity to change” coaching framework, which relies on bringing unconscious change-resistant beliefs into consciousness (Kegan & Lahey, 2009). Indeed, a recent review of approaches to improving mental well-being cites acquiring “an experiential understanding of one’s own psychological processes” as one of the basic pillars of mental training (Dahl et al., 2015, 2020).

Finally, practitioners of these methods routinely report success in this endeavour. Both therapy clients and mindfulness trainees report improved awareness of their mental processes (Castonguay & Hill, 2006; Goldstein, 2017; Hill & Knox, 2008; Kabat-Zinn, 1994; Young, 2016), often as a result of improved attention (Bernstein & Zvielli, 2014; Brown et al., 1984; Chan & Woollacott, 2007; Fox et al., 2012; Jha et al., 2007; Lutz et al., 2008; MacLean et al., 2010; Segal et al., 2018; Slagter et al., 2007; Tang et al., 2007). And the increase in self-reported awareness often mediates other beneficial effects of these practices (Castonguay & Hill, 2006; Ghasemipour et al., 2013; Hanley & Garland, 2017; Nakajima et al., 2017, 2019; Nyklíček et al., 2020).

Of course, these applied frameworks claim to involve much more than just improving awareness of internal processes. And there are certainly approaches to mental improvement which do not emphasize internal awareness; for instance, some clinical researchers argue that therapy gives people useful (but not necessarily veridical) beliefs or narratives about their mental processes without granting actual conscious access to those processes (McAdams, 1997; Wampold et al., 2007). Nonetheless, becoming conscious of previously-unconscious mental events is thought to be an essential component of a diverse, widespread set of successful applied interventions, and IIB is a foundational theoretical assumption in many approaches to mental improvement (Dahl et al., 2015, 2020).

### Objective Evidence?

Unfortunately, aggregated anecdotes do not sum to good evidence. For such a widespread and foundational premise, IIB has received strikingly little direct experimental investigation.

The existing line of experimental work that comes closest to suggesting IIB is on people’s awareness of their implicit attitudes. Implicit attitudes were traditionally conceptualized as evaluative representations that activate and influence people without awareness (Greenwald & Banaji, 1995), as evidenced by the surprise and denial people commonly express when confronted with evidence of them (Monteith et al., 2001). Yet Hahn et al. (2014) found that, when cued to attend those attitudes, people start being able to report them accurately (as measured by subsequent performance on an Implicit Association Test). Moreover, people seem to not have been fully aware of those attitudes before attending to them; the experience of attending to and reporting them increases people’s explicit acknowledgements of bias, and leads to higher explicit-implicit attitude correlations (Hahn & Gawronski, 2019). Thus, implicit attitudes seem like a candidate for a mental representation subject to IIB, which can be brought into awareness via attention (Hahn & Goedderz, 2020; Morris & Kurdi, 2023). (This possibility is bolstered by the fact that people with mindfulness/meditation training show less of a divergence between their implicit and explicit attitudes (Carlson, 2013; Koole et al., 2009; Remmers et al., 2018; Strick & Papies, 2017), and that people, when induced to attend to themselves, report attitudes more consistent with their subsequent behavior (Gibbons, 1983).

A second suggestive line of research comes from Carpenter et al. (2019), who tested whether people can improve at recognizing the accuracy of their own judgments. Participants performed a perceptual discrimination task, gave a metacognitive judgment of their accuracy on each trial (“how confident are you that you got it right?”), and then received real-time feedback on the calibration of those metacognitive judgments (Fleming et al., 2010). This feedback improved their metacognitive accuracy in both the perceptual discrimination task and a subsequent memory recognition task, suggesting domain-general improvement in metacognitive judgment through training. This improvement could potentially be the result of increased conscious access to the processes underlying perceptual discrimination or memory recognition (Morales, 2024; Morales et al., 2018). (It’s also possible, however, that the training improved people’s ability to infer their accuracy based on some other mechanism, like self-observation or inference; Hampton, 2009; Reder & Schunn, 2014.) This finding is echoed by Baird et al. (2014), who found that meditation training improves some forms of metacognitive judgment.

A third suggestive line of research comes from attempts to train internal awareness directly via coaching or guided interviews (Hurlburt & Heavey, 2001; Petitmengin, 2006). In these procedures, trained coaches guide participants through reporting their internal experience, helping them avoid pitfalls and focusing their attention directly on their mental events. Examples include the “elicitation interview” method from the neurophenomenological tradition (Petitmengin, 2006; Varela, 1996), and the “descriptive experience sampling” method (Hurlburt & Schwitzgebel, 2011). These procedures provide suggestive evidence for the possibility of bringing unconscious internal experiences into awareness. For instance, people who undergo guided interviews exhibit less choice blindness (a phenomenon plausibly involving introspective failure; Johansson et al., 2005; Petitmengin et al., 2013); show greater correspondence between reports of mental phenomena (like inner speech) and related neural activity (Hurlburt et al., 2016; Kühn et al., 2014; Lutz et al., 2002); and, among epileptics, report that they can better recognize and predict upcoming seizures (Petitmengin et al., 2006).

These scattered experimental results are consistent with the reports of practitioners that some mental events are unconscious due to inattention and can be brought into awareness if attended to. But this evidence is very limited, and is suggestive at best. People’s self-reports could of course be mistaken or confabulated; and the objective lines of evidence have only examined a very small swath of mental processes, and have not specifically tested whether attention is bringing unconscious mental events into awareness (as opposed to, e.g., improving people’s inferential abilities; for discussion, see Kurdi et al., 2024; Morris & Kurdi, 2023). As a hypothesis with foundational implications for both the theory and application of psychology, IIB demands more rigorous and systematic empirical treatment.

This lack of empirical investigation into IIB may be due, in part, to the difficulties of conceptualizing and testing for it. In the next two sections, I address some of these conceptual difficulties and then illustrate how the IIB hypothesis could be tested rigorously.

## CONCEPTUAL CLARIFICATIONS FOR THE IIB HYPOTHESIS

### The Scope of IIB

IIB has a potentially huge scope. If it is real—and some unconscious mental processes can indeed be brought into awareness via attention—then it is an open empirical question which processes, or which aspects of those processes, can be made conscious.

We can, of course, posit some sensible boundaries. There is little reason to suspect that people could ever become aware of low-level perceptual processes, like the computations underlying depth perception or the representations activated in V1; these internal events are likely permanently unconscious (Fodor, 1983). Moreover, awareness of a process is not all or nothing; even if a person can become aware of some high-level components of a process, there are low-level components which, intuitively, seem out of consciousness’s reach. For instance, even if people are conscious of performing mental rotation, it seems out of reach to introspect on low-level details of the computations which enabled that process (like the geometric rotation algorithm used, or the neurocognitive architecture underlying maintenance of the mental image).

On the flip side, the likeliest candidates for being successfully brought into awareness are high-level processes (and high-level components of those processes): those underlying judgment and decision-making, social cognition, inference, belief and attitude formation, etc. Given the breadth of unconscious high-level cognition, this potential scope is enormous (Hassin et al., 2006).

Moreover, even if people can only access high-level components of those processes (and not low-level implementational details), this would still constitute substantial awareness. For instance, consider a person who is initially unaware of how she is making frequency judgments, and then later reports that she is estimating the frequency of events (e.g., homicide) by observing the ease with which she can generate examples of them. Even without observing lower-level details of this process (e.g., the neurocognitive architecture implementing her memory retrieval), she has substantially improved her awareness of it—enough to describe it in the same level of detail that it was originally described by researchers (Tversky & Kahneman, 1973). For many high-level mental processes, substantial awareness seems possible without low-level knowledge of algorithmic details.^6^

Of course, the distinction here between “high-level” and “low-level” is vague. If there is a joint in the mind between preconscious and subliminal mental events, a precise characterization of it will have to await further research.

### The Relationship Between IIB, Metacognition, and Different Accounts of Consciousness

In this paper, I’ve adopted the access consciousness/global workspace account of conscious awareness as a useful framework for articulating the IIB hypothesis (Baars, 2002; Dehaene, 2014). But the possibility of IIB is compatible with other notions or theories of consciousness. For instance, when a person is described as conscious of something (e.g., a visual stimulus), that statement is often meant to indicate that the person has qualia or phenomenal experience of the thing (Block, 1995; Nagel, 1980; Tononi et al., 2016). The IIB hypothesis could be framed as internal attention bringing mental events into phenomenal experience. (Alternately, on theories where phenomenal experience “overflows” cognitive access, IIB could be framed as internal attention bringing mental events from phenomenal consciousness into access consciousness; Block, 2007, 2011; Lamme, 2006.)

On the other hand, the conscious awareness of mental events posited by the IIB hypothesis *is* importantly different from another related sense of awareness: metacognitive awareness (Dehaene et al., 2017). Metacognition is cognition about cognition: e.g., representing that you know some information, or forming a “second-order” representation about how accurate a “first-order” representation is (Fleming et al., 2012; Lau & Rosenthal, 2011; Morales et al., 2018). Common metacognitive assays, for instance, ask people to make perceptual judgments (“are these two low-contrast Gabor patches identical?”) and then make meta-judgments about how accurate their first-order judgment was (“how confident are you that you were right?”; Fleming & Lau, 2014). A rich literature demonstrates that people often possess metacognitive knowledge about their mental processes (Fleming & Frith, 2014).

This metacognitive knowledge, however, is conceptually separable from conscious awareness (in the sense used here). People could have metacognitive knowledge about their mental processes without having direct conscious awareness of them. For instance, a person who reads *Thinking Fast and Slow* may acquire metacognitive knowledge about how they make frequency judgments (e.g., representations of the form “I make frequency judgments via the availability heuristic”; Kahneman, 2011) without having introspected directly on that process. The same logic applies in the well-studied case of metacognitive confidence judgments. While it is possible that people’s confidence judgments are informed by conscious awareness of the underlying mental processes (Carpenter et al., 2019), it is also possible for people to be making accurate confidence judgments without having introspected on those processes (Hampton, 2009; Morales, 2024; Reder & Schunn, 2014). The IIB hypothesis states that people can gain direct conscious awareness of some mental events via internal attention (Dunne et al., 2019; Lutz et al., 2015)—not just that they can acquire metacognitive representations about those mental events.

Of course, metacognitive reasoning is still a crucial part of self-knowledge, (Fleming et al., 2010; Overgaard & Sørensen, 2004), and is likely deeply entangled with conscious awareness of mental processes. For instance, metacognition may facilitate, and be facilitated by, awareness of mental events (Brown et al., 2019; Lau & Rosenthal, 2011; Overgaard & Mogensen, 2017). Nonetheless, when theorizing about IIB, it is important to keep the two concepts distinct.^7^

### Obstacles to Overcoming IIB

In the external domain, overcoming inattentional blindness is easy. As soon as people are prompted to attend to, e.g., the invisible gorilla, they can bring it into awareness (Mack & Rock, 1998; Simons & Chabris, 1999).

In contrast, according to practitioner reports, the same is not true for internal events (Schwitzgebel, 2004, 2008). Even if bringing unconscious mental events into awareness is possible, it is allegedly quite difficult; few therapists or mindfulness teachers would simply instruct a client to pay attention to an internal process and expect immediate success. Rather, attending to internal processes is supposed to be a skill that takes time and effort to develop.

The difficulty of attending to mental events is vividly illustrated by the “guided interview” methods described above (Hurlburt & Schwitzgebel, 2011; Petitmengin, 2006). In these methods, a practitioner leads a subject through an interview about their experience at a moment in time, helping them attend carefully to their internal processes. It is a laborious process, often taking upwards of half an hour for the subject to describe their internal experience at one moment in time. And subjects are, at first, really bad at it; it is only with much coaching that subjects begin to report anything plausibly resembling their actual experience.

Why is it so hard? Anecdotally, when asked to introspect, people habitually do other things instead; they “flee from actual phenomena and distort or mask them in a variety of ways” (Hurlburt & Akhter, 2006). For instance, they do the things Nisbett and Wilson (1977) describe: They fall back on *a priori* theories or shared cultural suppositions, or make inferences about themselves. As Petitmengen puts it: “When asked to describe a given cognitive process, our natural tendency is to slip surreptitiously from the description of our actual experience toward the verbalization of justifications, beliefs, explanations, generalizations, and abstract knowledge about our experience” (Petitmengin et al., 2013). Accurately introspecting on mental processes requires learning to carefully sort through these layers of distortion.

Moreover, internal attention can be difficult to control with precision. The mental processes underlying judgments, choices, etc., happen extremely quickly, on the order of tenths or hundredths of a second; people do not have practice internally attending to events that fleeting. This problem is exacerbated by the fact that internal events are likely much weaker than typical perceptual targets of external attention (in the way that “seeing” a mental image is typically harder than seeing an actual image). Yet another obstacle is that, for people to focus on the mental processes underlying their behavior, they must attend internally to those processes while simultaneously attending to whatever they are doing externally—thereby splitting their attention and taxing cognitive resources even further (Lutz et al., 2015).^8^ It is unsurprising, then, that practices like mindfulness or cognitive therapy so often involve building attentional capacity (Lutz et al., 2008; Segal et al., 2018).

A final reason that IIB may be difficult to overcome is motivational: Uncovering unconscious processes can be an upsetting and identity-shaking experience, as the theories or rationalizations we have about ourselves often diverge from how we actually operate. People may get glimpses of their hidden processes and then quickly flinch away (Petitmengin et al., 2013). Moreover, when reporting on one’s mental processes in social contexts, there is often little incentive to report them truthfully; people could plausibly have developed a habit of giving introspective reports that please their social partners (Bem, 1967), rather than actually doing the work of orienting internal attention.

These difficulties may explain why people do not immediately start accurately reporting their mental processes when asked to attend to them (Nisbett & Wilson, 1977). (These obstacles also clarify how attending internally to a mental process in one’s own mind is very different from attending to or thinking about that process abstractly/scientifically—which is why experimental psychologists don’t automatically gain introspective insight into the processes they study.) Of course, much of this is anecdeotal, and precise answers may have to await a more developed account of internal attention training and IIB. But these reports suggest that simply instructing people to attend to a target mental process won’t be enough; people may need training.

## HOW TO TEST THE IIB HYPOTHESIS

Despite its potential importance (and its widespread assumption in applied frameworks), the possibility of IIB—and of bringing unconscious mental processes into awareness—has received very little direct empirical investigation with objective measurements. This lacuna may be in part due to perceived difficulty in testing for it.

**Figure 2.**
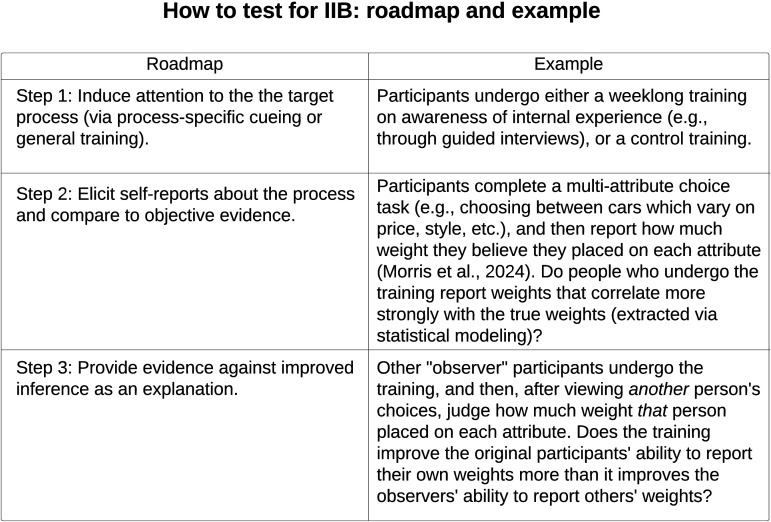
Conceptual roadmap of how to test whether a (component of) a mental process is unconscious due to internal inattentional blindness, and can be brought into awareness via attention. Any test must (1) induce people to attend to the target component of the mental process (by either cueing people to the process directly or providing general attentional training); (2) elicit people’s self-reports about the target process, and compare their reports to objective evidence; and (3) if the attention induction improves the match between people’s self-reports and the objective evidence, provide evidence that the improvement did not come from improved inference about the process (as posited by, e.g., Bem, 1967; Cooney & Gazzaniga, 2003; Nisbett & Wilson, 1977).

Here, I describe schematically how to test for it (Figure 2). Consider any mental process which research has discovered, but which people cannot accurately report. To test whether this process is actually preconscious due to IIB and can be brought into awareness, we need to induce people to better attend to their internal events (through process-specific cues or general training), and then measure whether they can more accurately report on the process in a way that most plausibly comes from increased conscious access to the process.

To take one of myriad potential examples, consider the “mere exposure effect”: the finding that repeated exposure to a novel stimulus makes people like it more (Zajonc, 1968). This effect is extremely robust, and yet people are largely unaware of it, typically denying an influence of familiarity and instead attributing their positive attitude to some irrelevant property of the familiar stimulus (Bornstein & Craver-Lemley, 2016). To test whether the internal events underlying this effect (e.g., the activation of a sense of “familiarity” while making liking judgments) are unconscious due to IIB, we would induce people to attend to those events and then test whether they can more accurately report the influence of familiarity on their judgments.

This simple description, of course, belies complex methodological issues. For one, we need to know how to induce people to internally attend to a target process—a feat which, according to practitioner reports, can be very difficult. We also need to objectively measure the accuracy of people’s self-reports about the process (Ericsson & Simon, 1984; Marti et al., 2010; Schwitzgebel, 2004; Shanks & St. John, 1994; Varela, 1996).

Most challengingly, we need to show that any improvements in reporting accuracy are due to increased conscious access to the mental process, as opposed to improved theory or inference about the process (Nisbett & Wilson, 1977). This issue is particularly delicate. We know that people make inferences and build complex theories about the underlying causes of people’s behavior, using observation, reasoning, and cultural knowledge (Kelley, 1967; Tenenbaum et al., 2006); and we know that they apply this cognitive machinery towards themselves, inferring their own mental processes through self-observation (Bem, 1972; Carruthers, 2009; Cooney & Gazzaniga, 2003; Cushman, 2020; Moutoussis et al., 2014; Wilson, 2004). Moreover, classic evidence against introspection revolves around this confound, showing that many apparent instances of accurate introspection are actually instances either of inference from self-observation or of “incidentally correct employment of *a priori* causal theories” (Nisbett & Wilson, 1977). The same problem applies here: In order to show that internal attention improves conscious awareness of a process, we need to show that increases in the accuracy of people’s self-reports are not due to improved inference or theory about the process.

I will discuss each issue in turn, with the goal, not to develop a specific experiment or overcome all possible methodological obstacles, but rather to illustrate that IIB is testable and provide a roadmap for how to do it.

### Inducing People to Attend to a Mental Process

The first challenge facing empirical tests of IIB is inducing people to attend to a target mental process. There are two basic approaches: directly cue people to attend to the target process/internal event, or train people to attend to internal events in a process-general way.

Past studies have employed both approaches. The studies by Hahn et al. (2014) on implicit attitude awareness, for instance, take the former approach; they describe what implicit attitudes are, direct people to attend to them, and measure people’s subsequent ability to report them accurately (Hahn & Gawronski, 2019; Hahn et al., 2014). The studies with guided coaching, in contrast, take the latter approach; they train people to attend broadly to their internal events (avoiding distractions, theorizing, and other common pitfalls of introspection), and then measure people’s subsequent accuracy at reporting things like internal speech (Hurlburt et al., 2016; Kühn et al., 2014).

In practice, most applied interventions combine the two approaches. For example, in therapies like CBT and DBT, patients practice attending to internal events in a domain-general way (e.g., through thought journaling), and also are cued to attend to specific thought patterns or emotional processes (Beck et al., 1979; Linehan, 1993; Segal et al., 2018). Similarly, meditators practice focusing attention in general, and are also cued to attend to specific sensations (like mental images or inner speech; Goldstein, 2017; Kabat-Zinn, 1994; Young, 2016).

What kinds of process-general training would be the best candidates for bringing unconscious processes into awareness? There are many practices involving internal attention training, and reviewing them all is outside the scope of this paper. Two particularly promising approaches would be meditation methods focused on non-judgmentally attending to internal cognitions (like noticing thoughts, mental images, inner speech, etc.; Young, 2016), and guided interview methods (Hurlburt et al., 2016; Hurlburt & Heavey, 2001; Petitmengin, 2006). There are also promising methods which train people by repeatedly giving them direct, immediate feedback on the accuracy of their internal attention and letting them learn implicitly how to control it (Amir et al., 2021; Bernstein & Zvielli, 2014; Ruimi et al., 2020). Which, if any, of these training methods is successful at reducing IIB is a question for future empirical work.

Motivated by the difficulties of directing internal attention, though, we can identify three features that any internal attention training should have to be successful. It should give people practice focusing their internal attention on increasingly subtle experiences while simultaneously attending to external tasks. It should teach them to notice when they are *not* attending to direct experience (when they are theorizing, making inferences, rationalizing, etc.), and to refocus. And it should help them overcome the motivation to turn away from unpleasant internal discoveries, perhaps by helping them observe themselves with equanimity or compassion (Kabat-Zinn, 2015; Young, 2016), or giving incentives for accuracy.

A goal for future research would be to demonstrate that such interventions are, in fact, improving internal attention. One way to do this would be to develop a measure of internal attentional capacity—an internal analog, perhaps of the attention network task (Fan et al., 2002; for some progress towards this, see van Ede et al., 2020). Researchers could then test whether the effect of interventions is mediated by increased internal attentional capacity.

### Measuring the Accuracy of People’s Self-Reports About a Process

The second challenge facing empirical tests of IIB is to determine whether the attention induction succeeded: whether people subsequently become more accurate at reporting their internal events or processes. This requires comparing people’s reports to objective evidence for the existence of an internal event/process, and testing whether people who receive an attention induction give reports that, on average, better match the objective evidence (Ericsson & Simon, 1984; Fleming & Lau, 2014; Marti et al., 2010; Shanks & St. John, 1994; Varela, 1996).

For instance, in a mere exposure effect paradigm, people’s liking judgments are influenced by familiarity with the stimuli, and yet people typically do not report this influence (Bornstein & Craver-Lemley, 2016). If people who undergo internal attention training subsequently report an influence of familiarity, this would suggest that the effect of familiarity can be brought into awareness via attention. Or another example: In multi-attribute choice paradigms (e.g., choosing between cars which vary simultaneously on price, looks, horsepower, gas mileage, etc), we can determine from people’s choices the weights they’re placing on each attribute (Bhatia & Stewart, 2018; Slovic & Lichtenstein, 1971). If people who undergo internal attention training report weights that correlate better with the observed weights, that would be evidence that their decision process can be made more conscious via attention (Carlson et al., 2023; Morris et al., 2023). These examples are two of many; since any mental process posited by experimental psychology has (presumably) been posited because of objective evidence, we can compare that evidence to people’s self-reports and measure whether awareness of the process improves with training.

Of course, this description leaves open many questions. For one, people’s ability to accurately report their mental processes may be confounded with other variables such as individual differences in the “strength” of those processes. This issue has been explored extensively in research on metacognitive confidence judgments (Fleming & Daw, 2017; Fleming & Lau, 2014; Rouault et al., 2018), and tests of IIB may be able to adapt some of those methods (such as using performance-controlled statistics like meta-*d*′; Fleming & Lau, 2014).

Moreover, there are questions about how best to query people’s awareness, a subject which has received much attention (Ericsson & Simon, 1984; Maia & McClelland, 2004; Newell & Shanks, 2014; Persaud et al., 2007; Shanks & St. John, 1994). Early work on introspective awareness used relatively crude measures; Nisbett and Wilson (1977), for instance, often simply described the target process to participants and asked them whether they had used it (for further criticism of Nisbett & Wilson’s methods, see Newell & Shanks, 2014; Smith & Miller, 1978; White, 1980). Since then, researchers have developed more careful measures of awareness, using techniques like think-aloud protocols (Ericsson & Simon, 1984), trial-by-trial queries (Lagnado et al., 2006; Maia & McClelland, 2004), wagering (Persaud et al., 2007), recognition tests (Reilly & Doherty, 1992), and more (Newell & Shanks, 2014; Timmermans & Cleeremans, 2015). These measures were developed largely to test whether specific processes—such as conditioning (Lovibond & Shanks, 2002; Olson & Fazio, 2001; Shanks & St. John, 1994), multiple-cue learning (Evans et al., 2003; Lagnado et al., 2006), and multi-attribute choice (Carlson et al., 2023; Gavanski & Hoffman, 1987; Harries et al., 2000; Maia & McClelland, 2004; Morris et al., 2023; Persaud et al., 2007; Reilly & Doherty, 1992)—are conscious by default. Nonetheless, the same techniques could be used to measure *improvements* in awareness, and to test whether high-level unconscious processes can *become* conscious through training.

One nuance is whether to measure the accuracy of people’s self-reports at the individual or group level. It is easier to experimentally establish the existence of a mental process at a group level—i.e., to show that people, on average, like familiar stimuli more than unfamiliar ones (Zajonc, 1968), or use “ease of generating examples” as a factor in frequency judgment (Tversky & Kahneman, 1973). It is more difficult to prove what happened in any one person’s mind. The existence of many processes can only be robustly observed in between-subjects manipulations (Charness et al., 2012); and even for effects that can be observed in within-subject manipulations, it is hard to determine whether the target mental process definitely occurred in that individual. Of course, there are contexts where we can get good objective evidence about what’s going on in an individual’s mind. For instance, we can identify the weights a person places on attributes in multi-attribute choice (Bhatia & Stewart, 2018; Carlson et al., 2023; Morris et al., 2023; Slovic & Lichtenstein, 1971; Smith & Miller, 1978); measure a person’s implicit attitudes (Hahn et al., 2014); or use process-tracing methods (like eye-tracking) to gain more fine-grained information about the person’s process (Schulte-Mecklenbeck et al., 2011). But establishing that a mental process happened in an individual will inevitably be noisier and harder than establishing that a mental process happens on average.

Fortunately, both types of evidence—individual and group level—can be used to test for IIB. In contexts where we can get individual-level evidence for the existence of an internal process/event, we can test whether internal attention improves the match between each individual’s process and their self-report of that process (e.g., testing whether training improves the correlation between self-reported and observed attribute weights in a multi-attribute choice task; Carlson et al., 2023; Morris et al., 2023). And in contexts where we only know that a process happens on average, we can test whether internal attention improves people’s ability, on average, to accurately report the process we know to be occurring on average (e.g., testing whether training improves the percentage of people who report using “ease of generating examples” to make frequency judgments). These approaches can be complementary, with individual-level tests being more precise but group-level tests enabling an examination of more diverse processes.

Finally, an additional obstacle in this step is that there is rarely consensus on the nature of psychological processes; how do we test for improvements in people’s introspective accuracy when we as scientists cannot agree on the processes occurring in their minds? The approach advocated here is to start by testing, not entire processes, but *aspects* or components of processes whose nature we can confidently characterize empirically. For instance, rather than asking people to report the entire process underlying their stimulus evaluations, we can probe specifically for whether they are aware of the influence of familiarity (i.e., in the mere exposure effect). Notably, even if our best theories only offer an approximation to the actual mental processes occurring in people’s minds, those approximations are likely closer to the ground truth than are people’s baseline self-reports in most domains (Hassin et al., 2006; Nisbett & Wilson, 1977). If, after internal attentional training, people’s reports of their mental processes better match our best scientific accounts, the most plausible explanation is that people have indeed gotten more accurate.

### Showing That Improvements in Introspective Accuracy Are Due to Increased Conscious Access, Rather Than Just Improved Inference or Theorizing

A third challenge facing any experimental test of IIB is to show that accurate reporting of the target process is due to increased conscious access to the process, rather than just improved inference or theorizing about it. As described above, people have lay theories about how their minds work, and can make inferences about their mental processes without having conscious access to those processes (Bem, 1967; Carruthers, 2009; Cooney & Gazzaniga, 2003; Cushman, 2020; Gazzaniga, 2005; Gopnik, 1993; Nisbett & Wilson, 1977; Wilson, 2004). Nisbett and Wilson (1977) rightfully argued that, to claim people have introspective access to a process, a central challenge is to show that accurate reports about the process didn’t just come from this kind of self-observation or inference. The same logic applies here: To claim that people can *gain* introspective access to a process, we need to show that *improvements* in accurate reporting did not just come from better self-observation or inference.

Though an inference account is difficult to completely rule out, there are several ways to provide evidence against it. One approach would be to use attention inductions whose content is completely unrelated to the target process. It is not obvious how a course on mindfulness meditation, for example, would provide new observations sufficient to infer that familiarity breeds liking in a mere exposure paradigm, or to infer the weight placed on “gas mileage” in a multi-attribute choice task. (Even if such interventions did generally improve people’s ability to perform self-observation/inference—e.g., by monitoring their own bodily reactions—these improvements would likely be insufficient to give people new, fine-grained knowledge about their mental processes.) So if these interventions successfully improved reporting accuracy, improved inference would be an unlikely explanation.

Another approach would be to test for awareness of aspects of the target process that would be difficult to infer without conscious access. For instance, the mere exposure effect does not occur when the stimuli presentations are clumped together homogenously (i.e., when all presentations of a stimulus occur consecutively; Bornstein & Craver-Lemley, 2016). This boundary condition would be extremely difficult to infer from lay theorizing; yet, a person who attained conscious access to the process underlying their liking judgments would be able to correctly report that familiarity did not, in that context, play a role.

Finally, researchers could adopt the suggestion of Nisbett and Wilson (1977): Compare people’s self-reports to the inferences of observers. Nisbett and Wilson (1977) recommended giving “observer” participants a vague description of the task and seeing if they can infer people’s mental processes as well as the people themselves. Researchers could go a step further by matching each observer with an actual participant, and giving the observer a description of as many observable facts about the participant as possible: their full experience of the task, their demographic information (Morris & Kurdi, 2023), even a video of them performing the task. Participants and observers could both receive the attention induction. If the induction improves people’s ability to report their own process more than it improves observers’ ability to infer another person’s process, that would provide further evidence that internally attending to the process improves accuracy by bringing it into conscious awareness (Gavanski & Hoffman, 1987; Morris et al., 2023).

Of course, even using the most stringent controls, it would be difficult to rule out that the training improved people’s motivation or ability to infer their own mental processes through some internal self-observation technique that is different from gaining conscious access to the process itself. More generally, even if internal attentional trainings did yield increased conscious access to mental processes, it is likely that the intervening mechanisms would be multi-faceted. Maybe sometimes people would become aware of the process immediately upon attending to it; but other times, maybe people would have a vague memory of the process which the increased attention helped them remember or articulate more clearly (see the discussion of inattentional blindness vs amnesia or agnosia in Inattentional Blindness Towards Internal Events section above). These fine-grained distinctions matter theoretically, and disentangling them is an important direction for future research. But, zooming out, they start to become less important. If internal attentional training substantially improves people’s ability to accurately report on their unconscious processes in a way that observers cannot, this finding would suggest that people can learn to access a “fount of privileged knowledge” (Bem, 1967) about their own mental processes, and would significantly advance our understanding of the potential of introspection.

## IMPLICATIONS OF THE IIB HYPOTHESIS

If applied practitioners are right, and important high-level mental processes can be demonstrably brought into awareness via attention, this finding would have significant ramifications for basic experimental psychology.

For one, it would have implications for a fundamental question about the mind: how much of our own mental lives we can become conscious of. This question, which has been spotlighted throughout the history of psychology (Mandler, 2011; Schwitzgebel, 2004, 2008; Wilson, 2004), shows up in a diverse array of influential contemporary theories. It shows up directly in the work of Nisbett and Wilson (1977), who argued that people have little introspective access to their mental processes “no matter how hard we try” (Nisbett & Wilson, 1977; Wilson, 2004). But similar sentiments show up indirectly in other accounts. In the introduction to his bestseller *Thinking Fast & Slow*, Kahneman (2011, p. 2) writes:

When you are asked what you are thinking about, you can normally answer. You believe you know what goes on in your mind, which often consists of one conscious thought leading in an orderly way to another. But that is not the only way the mind works, nor indeed is that the typical way. Most impressions and thoughts arise in your conscious experience without your knowing how they got there. … The mental work that produces impressions, intuitions, and many decisions goes on in silence in our mind.

A similar dual-process account is given by Haidt (2012, p. xxi) in his bestseller *The Righteous Mind*:

The rider is our conscious reasoning—the stream of words and images of which we are fully aware. The elephant is the other 99 percent of mental processes—the ones that occur outside of awareness but that actually govern most of our behavior.

Dual-process accounts, like those proposed by Kahneman, Haidt, and others, do not depend on “System 1” being permanently unconscious (Evans, 2003). Nonetheless, these introductions would have to be rewritten if it turned out that people, with training, could become directly conscious of many of the hidden processes producing their intuitions, judgments, and choices. The same applies to theories of implicit attitudes (Banaji & Greenwald, 2016; Hahn et al., 2014), automatic social cognition (Bargh, 2013; Bargh & Morsella, 2008; Bargh & Williams, 2006), moral psychology (Greene, 2013; Haidt, 2001), somatic markers (Bechara et al., 1997), and more (Dunning, 2012; Gigerenzer, 2008; Hassin et al., 2006). Moreover, many of the other features posited to be inherent to these processes—such as their automaticity or uncontrollability (Evans, 2003, 2008)—could plausibly change if the processes are brought into consciousness.^9^

The IIB hypothesis, if it were true, would also have implications for accounts of “cognitive illusions” (Pohl, 2016). For instance, consider the “illusion of conscious will” (Wegner, 2002). This influential account posits that people’s sense of consciously willing an action is dissociable from the actual mental events producing the action (Libet, 1985; Wegner, 2003; though see Schurger et al., 2021 for alternate views). Building on these findings, Wegner (2002, 2003) argues that people do not access veridical representations of intention or action initiation, but instead infer conscious causation post-hoc—i.e., they experience an illusion of conscious will. Though Wegner’s account does not depend on the illusion being permanent, the interpretation of these data would be quite different if people stopped showing the illusion after training. The same is true for theories of choice blindness (Johansson et al., 2005; Petitmengin et al., 2013), constructed preferences (Slovic, 1995), and other cognitive illusions (Chater, 2018; Pohl, 2016).^10^

Past challenges to the purported unconsciousness of high-level cognition have focused on whether high-level processes are, in fact, unconscious—by arguing that demonstrations of unconsciousness have failed to accurately measure awareness (Newell & Shanks, 2014; Smith & Miller, 1978; White, 1980), or identifying processes of which people do seem aware (Ericsson & Simon, 1984; Lagnado et al., 2006; Lovibond & Shanks, 2002; Maia & McClelland, 2004). Where the line falls between conscious and unconscious cognition continues to be hotly debated (Chater, 2018; Newell & Shanks, 2023; Shanks & Newell, 2014). But this debate largely ignores the possibility that the dividing line is labile—that it is attention, not structural constraints, which often determine whether a process is conscious. If the IIB hypothesis is right, it would suggest that the debate should be framed, not just as which processes *are* by default conscious, but also as which *can be made* conscious or not.

Despite the potentially-widespread theoretical impact of the IIB hypothesis, it has received almost no direct investigation. Nearly all the tests for introspection into high-level cognition use participants who have not been trained in it (Hurlburt & Heavey, 2001; Newell & Shanks, 2014; Nisbett & Wilson, 1977; Petitmengin, 2006). But if practitioner reports are to be taken seriously, this evidence is highly inconclusive; it would be akin to concluding that mathematical truths are inherently inaccessible because eight-year-olds cannot comprehend calculus. Another way to put it is that this accessible-or-not dichotomy is not true of external perception; external perception is highly dependent on attention. There are some things that are permanently unconscious and others that are easy to see, but many stimuli are in the middle—you only become conscious of them if you look carefully. The same is plausibly true for internal perception—and doubly so, since the internal cues are inherently weaker and internal attention is plausibly more difficult to control. Accounts which invoke the scope of consciousness in the mind cannot afford to ignore the possibility of IIB, and of a labile, skill-based boundary between conscious and unconscious.

On the flip side, applied practices like cognitive therapy and mindfulness training cannot afford to go on assuming the existence of IIB without testing it. These practices are enormously popular; 50% of American households have someone visit a therapist each year (Chamberlin, 2004), over 40% of Americans report meditating at least once a week (Masci & Hackett, 2018), and there is a burgeoning industry of other mental training practices aimed, in part, at improving “experiential access” to mental processes (Dahl et al., 2020). Though there is much evidence for the overall efficacy of these techniques, their mechanistic claims outpace their evidence base; they report bringing unconscious processes into awareness, but almost never test those claims objectively. If the IIB hypothesis is right, it could offer a cognitively-grounded framework for developing, improving, and validating introspection trainings. And if it is wrong, then a key aspect of these practices must be reconsidered.

Finally, as discussed above, if the IIB hypothesis is right, it could impact the practice of psychological research. It is a poorly-kept secret that much of our science is guided by scientists’ introspection. To quote Jack and Roepstorff (2003, p. vii):

An informal reliance on introspective evidence is ubiquitous in psychology and cognitive science. It generates many of the hypotheses that psychologists seek to test using objective sources of evidence, it underlies their understanding of cognitive tasks or ‘task analysis’, and it frequently informs the questions and objections they offer as referees. Introspective understanding even forms the basis of many of the categories used to describe branches of psychological research (e.g., ‘attention’, ‘episodic memory’, ‘awareness’).

Even though our science does not (and should not) rest on introspective evidence, it is often informally guided by it (Cowan & Rachev, 2018; Locke, 2009; Schooler, 2002a).^11^ Just as turning on more lights could dramatically accelerate the pace of finding your keys, expanding conscious access to mental processes could significantly speed up the pace of psychological discovery.

## CONCLUSION

Just as people can miss perceptual events due to external inattention, so may they miss internal events—like those constituting high-level mental processes—due to internal inattention. The existence of internal inattentional blindness, and the possibility of overcoming it through training, are widely assumed in applied psychological practices and widely reported by practitioners; yet these possibilities have rarely been explored experimentally, or taken seriously by basic theorists. Rigorously testing the existence of IIB could open a new chapter both in the development of psychological interventions, and in our understanding of the scope of conscious awareness.

## Acknowledgments

Thanks to Fiery Cushman, Molly Crockett, Ryan Carlson, Hedy Kober, Adam Bear, Josh Greene, Sydney Levine, Adina Roskies, and all the members of the Moral Psychology Research Lab and the Crockett Lab for their invaluable feedback and assistance.

## Funding Information

Adam Morris is supported by NIH postdoctoral fellowship 1F32MH131253.

## Notes

^1^ Similar sentiments have been expressed in other influential work (Bargh & Williams, 2006; Bechara et al., 1997; Carruthers, 2009; Dunning, 2012; Gigerenzer, 2008; Gopnik, 1993; Greenwald & Banaji, 1995; Haidt, 2012; Johansson et al., 2005; Kahneman, 2011; Schwitzgebel, 2008; Wegner, 2002), though see Newell and Shanks (2014) and Chater (2018) for alternate views.^2^ This question is related to debates about whether consciousness is graded vs. all-or-nothing; see Dehaene et al. (2014), Overgaard et al. (2006), Sergent and Dehaene (2004), and Windey et al. (2014).^3^ There is a trivial sense in which a great number of internal representations might be considered “preconscious”: all the latent memories, plans, images, etc., which you currently aren’t calling to mind but could at will. But this is a different sense of the term. Following Dehaene (2014), we restrict the label “preconscious” to representations which are *currently activated* (not latent), and which are not currently in consciousness but would enter it if attended to. (In other words, preconscious representations must be represented “by firing”, not “by wiring”.)^4^ In this paper I focus on people’s potential to become aware of symbolic mental processes. Of course, it is also possible that people could attend to and become aware of non-symbolic mental processes, and the arguments here may also apply in those cases.^5^ It is sometimes debated what counts as a “mental process” proper, versus merely the inputs/outputs of a mental process (Ericsson & Simon, 1984; Nisbett & Wilson, 1977; Smith & Miller, 1978; White, 1980). I use the term “mental process” broadly, to capture the process of representations being formed and transformed as sensory input is translated to behavioral output. If one wishes to use the term more narrowly—e.g., to only refer to algorithms at lower levels of the abstraction hierarchy—then the IIB hypothesis can be thought of as applying primarily to high-level mental events rather than mental processes.^6^ If the IIB hypothesis is right, then it is also an open empirical question how “deep” within a process conscious access can get. For instance, consider the process underlying multi-attribute choice—e.g., choosing between cars which vary simultaneously on price, horsepower, style, etc. In some contexts, people appear to weigh and combine the attributes linearly (Bhatia & Stewart, 2018); in other contexts, people appear to use nonlinear heuristics (such as choosing based on a single attribute (Gigerenzer et al., 1999), or eliminating items that don’t have certain attributes (Tversky, 1972)). Making this distinction involves detailed understanding of the underlying decision algorithm. And yet, if the IIB hypothesis is right, it seems plausible that people could become conscious of this distinction; someone could accurately observe, for instance, that they weighed together seven different attributes when selecting a car, but then chose solely based on price when selecting a beer (Carlson et al., 2023; Morris et al., 2023). The scope of internal attention and conscious access is something that needs to be determined empirically.^7^ Some higher-order theories of consciousness posit that certain kinds of metacognitive representations are necessary for, or constitutive of, conscious awareness (Brown et al., 2019; Lau & Rosenthal, 2011). In this case, the IIB hypothesis is that internal attention may help create those kinds of metacognitive representations of mental processes, and thus bring those processes into awareness. Even in this case, though, it would be important to distinguish between those awareness-granting kinds of metacognitive representations and ones that can be acquired, e.g., from reading a psychology book (Carruthers & Gennaro, 2023).^8^ Dividing attention between external tasks is hard, and often requires intensive training; people take upwards of 30 hours of practice, for instance, to become proficient at reading stories while transcribing words being spoken aloud (Hirst et al., 1980; Spelke et al., 1976). It is likely similarly difficult to divide attention between external and internal targets.^9^ If the mental process changes upon being brought into awareness, one could ask whether it is then the same process or not (and thus whether the IIB hypothesis—that some mental processes can be brought into awareness via internal attention—is correct). Philosophical discussion of this issue is outside the scope of this paper, but I will note that most substantial changes are likely to occur some time *after* the process is brought into awareness. For instance, if someone becomes aware that they are making frequency judgments with the availability heuristic and then chooses to stop using the heuristic, that choice likely happens well after the heuristic came into awareness. Hence, it is appropriate to say that internal attention brought the availability heuristic itself into awareness, after which it was modified.^10^ Another set of psychological accounts that would be impacted by the existence of genuine introspection training are ones that rely largely on self-report: for instance, positive psychology (Peterson, 2006), personality psychology (Paulhus & Vazire, 2007), symptom-focused clinical psychology, and studies of explicit attitudes (Ajzen, 1991). These disciplines often rely on people’s ability to report features of their mental lives—their happiness, dispositions, attitudes, internal experiences, and so on. If people can become provably better at noticing and reporting internal events, they could plausibly learn to answer these questions more accurately.^11^ Anecdotally, Kahneman claims to have developed many of his Nobel-prize winning theories in part by introspecting on his own judgment and decision processes (Kahneman, 2011).
